# Facts and Observations in Medicine and Surgery

**Published:** 1845-01

**Authors:** 


Facts and Observations in Medicine and Surgery. By *John Grantham.* Octavo, pp. 216. Churchill, 1844.

The Facts and Observations contained in this work have appeared from time to time in the Medical Gazette, and we question the author's policy in transferring them from the small type and double column of our cotemporary to the present broad-margined smart-looking volume. Passing muster very well amidst the miscellaneous matters of a periodical, collected apart contributions of this sort are so often disappointing as to be seldom read, unless when known to be the productions of esteemed writers. We fear this work will prove no exception to the general rule: for, although several of the facts are interesting, they are not numerous or important enough to constitute a substantive volume, while many of the author's observations upon disease are vague and verbose, and some of the extracts with which he has interlarded them of undue length and insufficient bearing.

We will now refer to those passages we consider most interesting.

*Treatment of Fractures by Suspension.*—Mr. Grantham considers the advantages attending the treatment of fractures of the lower extremity by suspension have not been duly appreciated in this country. He translates a paper by Mayor of Geneva, detailing some of these, and corroborates the conclusions arrived at by the results of his own practice. The limb, inclosed in splints as usual, is not allowed to lie upon the bed, impeding every movement of the patient, and becoming deranged in the apposition of its fractured parts, or otherwise injured, whenever the posture is changed. Protected by a pad, it is laid upon a wooden frame, and the whole suspended by ropes extending from the ceiling or upper part of the bed underneath the apparatus. So adjusted, the movements of the patient are much less restrained; for, in ordinary cases, he may sit out of bed daily, and in the case of bad compound fractures, quietude of the parts is often obtained to the extent of allowing the patient refreshing sleep; and, indeed, many limbs, which under ordinary treatment would have been condemned to amputation are preserved to the patient. Union, too, takes place with great rapidity, while less callus than ordinary is deposited. Dr. Wigan is quoted as having observed the excellent effects of this practice: he says :—

"I saw it employed in many cases at Lausanne, and was the means of introducing it into some parts of Italy and France; but although I had often seen patients get in and out of bed without any assistance, and without risk of disturbing the limb, and spared no asseverations to convince, it was rare that I could get any one to comprehend fully the extreme simplicity and perfection of the apparatus, and I was often unsuccessful. I have observed all over Europe, that surgeons are miserable mechanics, and have seen many apparatus by common bone-setters, Greeks, Germans, and Dutch, which would shame our hospital surgeons.

"In passing from Lausanne into Italy, I found a lady at a miserable cottage in the High Alps, who had been six weeks on the floor, having broken her leg by the fall of a mule, in passing over the Wengern Alp. Serious injury was coming on in various parts of the body, from confinement to one position. On the application of the new apparatus, she was immediately able to rise: her gratitude was unbounded, and her recovery so rapid, that a few weeks afterwards I encountered her at Milan, quite restored.

"Dr. Mayor never claimed the merit of the invention, but he made great efforts to teach the peasantry of the mountains the use of the apparatus, which was within the means of any one to construct—a matter of importance in their insulated life, where they could not procure surgical aid at any price for five or six months together.

"The mode of fastening which I saw in Switzerland, was rather clumsy and complicated. I substituted the *guy*, which I think is an improvement." 35.

The author relates but three of the cases to which he has applied this plan of treatment, but there can be no doubt it is deserving of far more notice in our large hospitals than it has hitherto received. The following is one of the cases.

"Copley, a bricklayer's labourer, bearing a hod of bricks on his shoulder, fell from a scaffold ten feet from the ground. He dislocated the ankle-joint inwards, and fractured the fibula at about three inches above its lower end. On the following day, I placed the limb on the suspension apparatus. The patient was assisted out of bed, and sat in a chair, with the fractured limb suspended by a cord attached to the ceiling. It is now the 25th day: ossific union has taken place; there is no tenderness of the joint, neither has he suffered severe pain, although there was much tumefaction and extravasation. He has not been confined to the bed one whole day since the accident occurred."

*Local Applications to Fractured and Contused Limbs.*—Mr. Grantham ojects strongly to the employment of cold applications, not only in the case of fractures, but in all injuries producing inflammatory action in the cartilaginous, fibrous, and muscular structures.

"It is now eight years since I first advocated the necessity of attending to and keeping up the action and temperature of the cutaneous structures in inflammation, laceration, or contusion of the cartilaginous, ligamentous, tendinous, fibrous, and muscular structures of the body, and also since I denounced in those injuries the application of cold, and the diminution of power by means of local bleeding near the wounded part. Experience has fully attested the many advantages of hot, stimulating, applications: they have a decided pre-eminence over all others in lessening the sufferings of the patient; while, on the other hand, much time is gained in restoring the reparative action of the parts, notwithstanding that tumefaction, redness, and swelling of the cutaneous and subcutaneous structures generally proceed with great activity during the first four days, then gradually subside about the seventh or eighth day, leaving the deep-seated structures comparatively free from pain either on motion or pressure; and the truth of this will appear evident in reflecting upon the well-known Hunterian law, that no two inflammations can exist with equal force in the same system, the one will mitigate the other. Next, we must consider the lowly organized state of these structures, which are only balanced or supported in their normal action or temperature by the power of the exhalent vessels of the skin. Did these lower structures contain but one drachm of blood more than in their normal condition, the result would be inflammation, which would require at least three months to effect a termination by resolution. It is a fact that excitement of the arteries in the vicinity of an injury assists much in the restoration of a sanative action. Only diminish the power of the arteries and exhalents below the natural standard in such injuries, and you deprive the limb of the means of repairing the mischief, and too frequently bring on phlegmonous inflammation or sphacelus. By this statement I do not mean to condemn the necessity of general depletion when the pulse is quick and hard; but I feel bold in asserting, that local bleeding by leeches, with the application of cold lotions, prevents the well-doing of such cases." 68.

Two cases are related, one of dislocation of the hip, and the other of the elbow: the pain, tumefaction, and tenderness remaining after reduction in the former were dissipated by hot fomentations and mustard poultices, in the latter, by hot fomentations and stimulating embrocations. The author seems to us to resort too frequently to general bleeding in fracture as a *preventive* of inflammation. We would recommend some observations quoted from Mr. Travers in our present number to his notice.

*The Double-inclined Plane.*—" I beg leave to state my objection to the straight position in the treatment of fracture of the thigh and leg. From the commencement of my surgical life I have been in the habit of placing fractured limbs in such a position that the fewest muscles are rendered tense. In fractures of the thigh, notwithstanding the apparent simplicity and adaptability of Dessault's splint, I am practically convinced that the patient who is treated in such a manner loses much time during the convalescent stage. The bone may unite, the limb may be rendered straight by its application, but on a fair comparison with a proper double-inclined plane, rightly adjusted, [the author represents in a wood-cut the form of this he employs] the patient will suffer far less pain in getting the limb accommodated to that passive form, than by the extended position. Two things, therefore, are gained—a shorter duration of pain, and many weeks of time in the restoration of the muscular action during the convalescent stage of a fracture." 72.

*Extensive Burn.*—A case of a burn from gunpowder is related, in which nearly the whole of the trunk and a portion of the extremities were involved, " the whole measured above 600 superficial inches, or four feet,

24 inches, and averaged a quarter of an inch in depth. Also the subcutaneous structure was completely lost, so that the arteries and veins were seen, as if neatly dissected, lying on the surface of the muscles and fascia." The successful issue of the case reflects great credit upon the author, especially as more than one untoward occurrence intervened. Three principles especially guided him, the due supply of nutritive food, the regulation of the animal heat, and the external and internal use of antiseptic agents, such as the application of yeast, the evolution of oxymuriatic gas into the apartment, the administration of alkalis, &c. During the extensive suppuration which occurred, six pints of milk in the twenty-four hours served to support the youth's (æt. 17) strength. A sphacelated wound over the sacrum, an attack of bronchitis, and an extensive re-opening of the wound by erysipelas, successively retarded the cure, and long rendered recovery apparently hopeless. It required several years to produce entire healing, during and subsequently to which there has been much tendency to congestion of the brain, requiring small depletions and aperients, and attributable to the imperfect re-establishment of the functions of the skin over so large a surface.

*Insanity*.—Mr. Grantham has two chapters upon the premonitory symptoms and treatment of insanity. He says:—

"The duties of the general practitioner in cases of insanity, are chiefly limited to the treatment of the premonitory symptoms: it is at that time when the patient stands, as it were, on neutral ground, that the practitioner is sent for; at a time when he is surrounded by relatives and friends, and the soothing attentions of home—circumstances often the most favourable for the sufferer. But with these advantages, a very frequent cause of error in the judgment of a medical man (when called upon to decide as to the state and probable restoration of the patient) is the undue regard which is paid by him to the wishes and opinions of the friends, who seem to take a pleasure in enlarging upon the odd whims and irregularities of the patient. The fears of one party, and the ignorance of both, soon settle the question, and at once precipitate the poor sufferer from a state of comparative sanity into irremediable madness. A medical man will do well to prefer even the immethodical relations of the patient, rather than trust to the opinions of friends, for how often is it that actions, seemingly the most whimsical, may be strictly consistent with the physical condition of the invalid: thus insane persons, tormented with feverish heat, delight in exposing themselves to cold air or water, while, as M. Esquirol observes, a disordered state of the digestive organs often renders them averse to taking food." 113.

The importance of attention being paid to the early symptoms of insanity cannot be over-stated; but we must join issue with the author as regards the steps which should be taken upon a discovery of these. So far from having found friends anxious to make out the patient's case as worse than it is, and thus unnecessarily consign him to an asylum, we have too often observed the very contrary, an unwillingness to perceive that which has become obvious, and a delay in resorting to aid which has often proved detrimental to ultimate recovery. Moreover, the separation from home which he seems to regard as a calamity, is the step which is ordinarily essential to giving the patient a fair chance; and Esquirol and Pinel, whom he quotes as stating the greatest number of cures are effected within the first month, certainly are alluding to the case of patients so circumstanced.

To treat an hypochondriacal or hysterical patient (and many of the author's observations in this chapter are applicable to such) as insane would indeed be likely to aggravate their maladies, but when the diagnosis of insanity has been satisfactorily made out, seclusion cannot be accomplished too soon.

*Effects of Deficient Ossification of the Cranium.*—The following is part of a passage quoted from a paper upon the employment of compression in certain cases of *hydrocephalus*, which was contributed by Sir Gilbert Blane to the Med. and Phys. Journal for Oct. 1821.

" It occurred to me that the distension of the head and bregma is owing to a want of firmness and due resistance in the bony compages of the skull, which consequently yields to that effort of pressure with which the brain, in its growth, acts upon its parietes. In reasoning farther on the subject, it appeared to me conformable to some of the most approved principles of physiology, that, as there is a certain degree of tension and pressure necessary to the sound condition and action of parts, the withdrawing of this, by inviting afflux and congestion, produces serous effusion; and, for the like reason, there may be a deficiency of that interstitial absorption upon which the healthy state of this and of all other parts of the living frame depends."

The author adopts these views as explanatory of certain cases of epilepsy, cerebral congestion, and hydrocephalus; such imperfection of structure resulting from " improper nutriment, a strumous diathesis, and a lack of warmth of the skin during the first seven or eight months of infantile life." The cases of four infants are related, two affected with epileptic fits, one with " restlessness and general indisposition," and the other with " oppressed breathing and emaciation," in which the supporting the cranial bones by bandages seemed to be productive of benefit. From their small number, and the employment of other means of treatment likewise, they go for little.

*Galvanism.*—We quite agree with Mr. Grantham, that the application of galvanism in paralysis, and other chronic affections of the nervous system, has been too much neglected. Indeed, its administration seems to be confined almost to empirics, who apply it in all cases indiscriminately, and consequently do more harm than good. Why a full and fair trial of its medicinal powers should not be made in some of the numerous chronic cases which encumber the hospitals we cannot imagine. Mr. Grantham relates some cases in which he found this agent, carefully administered for a prolonged period, completely successful; and states the results of his experience in its employment in these conclusions.

" 1. Galvanism is identical with the vital action of the nerves of organic life, and the nerves of volition. 2. Its action is determined by the healthy condition of the brain and spinal marrow. 3. The skin must possess a normal sensation, as well as temperature, before the galvanic action can affect the muscular fibre. 4. The positive plate or wire should be applied over the region of the origin, and the negative to the region of the termination of the nerve. 5. The galvanic influence, when passed along the spine, will be most active in the paralyzed limb. 6. Galvanism is assisted by the alkalis and mercurial action. 7. It restores diminished temperature, decreased circulation, and lost muscular action, in the following order: 1st, temperature, 2nd, circulation, and muscular action last.

8. It has no effect in disease that alters the structure of nerves. 9. It supersedes manual friction. 10. It is assisted by immersion of the affected limb in a warm bath, into which the negative plate or wire is placed. In passing a current from the head through one half of the body, the foot should be immersed in warm water. 11. It is injurious when much pain is caused in the muscles by its application. 12. It may be carried to an undue extent, so as to produce congestion of the brain." 183.

*Secale Cornutum.*—Of this medicine the author thus speaks :—

"I have seen the uterus impaired, as to its future contractile power, after the use of large doses of ergot of rye, and have frequently had occasion, during the last two years, to apply the forceps to assist the parturient efforts of those persons in whom the practitioner had previously hastened the labour with the above drug. On the other hand, if the cases be properly selected, it forms a most useful auxiliary in effecting the expulsion of the fœtus. The æthereal tincture I have found very valuable in suppressing uterine hæmorrhage, and I am in the habit of giving one drachm, in a wine-glass of warm water, to mitigate the after-pain. It relieves the patient better than opium, and without producing any ill effect upon the sensorium. If too much discharge come on after delivery, I increase the dose to two drachms, and repeat it according to necessity." 195.

*Premature Vaccination.*—Mr. Grantham believes, from the greater insusceptibility of the infant constitution, this operation is best delayed until after the termination of the period of lactation, *i. e.* about the ninth month. He has come to this conclusion from observing that children vaccinated earlier are more liable afterwards to contract small-pox than those vaccinated at a later period. He quotes some observations, by Mr. Deane and Dr. Murray, of Cape Town, as confirmatory of the superior protective power of late vaccination. The last-mentioned experienced practitioner recommends the operation being delayed only to the 5th or 6th month. If a greater durability of the protective power really does result, the immediate consequences of delay need not excite much alarm; for young infants, however much exposed to infectious diseases, seldom contract them, and are sometimes the only members of the family who escape. When small-pox rages epidemically, however, no delay should be allowed, and we have seen very young infants infected, (and they usually die when they do contract the disease), during the epidemic of this last Summer and Autumn.

*Fibrinous Diarrhœa.*—The *Diarrhœa Tubularis* of Good. This is a rare and often an obstinately chronic disease. In all the cases which the author has seen, its production has been preceded by the exhibition of mercury, with the too active employment of aperients.

"In the cases that have come under my notice, the discharges from the intestines have assumed, in the first stages of this complaint, a mucous appearance; secondly, a mixed muco-fibrinous character; and lastly, the evacuations have contained true fibrin. The evacuations of fibrin have been preceded by long-continued pain in the abdomen, and great irregularity in the temperature of the skin; the individual being highly sensitive to a damp atmosphere, which caused spasmodic pains in the abdomen. When the mucous membrane of the fauces and posterior nares is implicated in the disease, the patient suffers much from violent pain in the head, referrible to the region of the parietal bones, with extreme irritability of mind. There is a great tendency to acidity in the stomach, which is increased under the use of a liquid diet. The abdominal pains are always of a spasmodic character, and at times very acute, extending to the neck of the bladder, and down the inner part of the thigh. The tongue assumes a white appearance, with indentations round the edges; sometimes ulceration of a phagadænic kind forms over the tonsils. The pulse is seldom altered, generally maintaining a steady, healthy, feel: the skin is often studded with numerous papulæ, especially over the chest, neck, and face. The urine denotes an anæmic condition of the kidneys: occasionally such patients pass urine with evident traces of albumen, seldom containing a normal quantity of the phosphates. On an increase of fever or mental excitement, a larger quantity of the lithate of ammonia is found: frequently the mucous membrane of the bladder is thickened in these cases. The fæces, which are very seldom incorporated with the fibrin, are often very healthy in appearance, clearly shewing a natural action of the glandular system. Although it is said by most authors that this disease is by no means fatal, yet I think I have seen it degenerate into atrophy of the intestines." 205.

Mr. Erasmus Wilson, writing to the author, after alluding to two cases which he had noted, and which occurred independently of the administration of mercury, observes—

"I do not agree with Dr. Golding Bird in thinking the principal seat of the inflammation in this disease to be the mucous follicles, nor does the false membrane appear to me to be simply an altered mucous secretion. The mucous follicles may, and very probably do, constantly participate in the inflammation of the mucous membrane: there is nothing anti-physiological, moreover, in supposing that they may be inflamed separately from the mucous membrane. But the character and appearance of the membranes that I have examined, their breadth and continuity, have led me to believe that they are a product of the superficial mucous surface. Animal chemistry can avail us little in determining the nature of a deposit of this kind. Protein, albumen, gelatine, fibrin, mucus—all are composed of the same elementary constituents, varying so slightly in proportion, that it is clear that they are all modifications of the same staminal principle. In croup, we do not hesitate to ascribe the production of the false membrane to the superficial mucous membrane; indeed, we have seeming proof that this is the case in the smaller relative proportion of the infected membrane, I am therefore desirous of claiming as much for the pulmonary mucous membrane in another place—namely, in the alimentary canal.

"Against my hypothesis of the analogy between croup and fibrinous diarrhœa, you might be inclined to urge the acute inflammation of the former, and the chronic character of the latter. But the latter is chronic only in duration. Its seat is limited and dispersed, and the patches which produce the lymph are each, as I imagine, as acutely inflamed as the pulmonary mucous membrane in croup." 215.

In the *treatment* of the disease Mr. Grantham recommends residence in a dry locality, warm clothing, friction and ablutions, regular exercise, with a mild, moderate, unstimulating diet. Purgatives must not be resorted to but as an occasional means of removing painful accumulations of fibrin, when castor oil is the best. A tepid enema, with or without narcotics, to be used every morning. Alkalis may be given according to the condition of the gastric secretions and the urine. In obstinate cases injections of nitrate of silver are useful; but stimulants, such as copaiba, turpentine, &c. are useless. In one bad case, cod's liver oil improved the condition of the patient without curing the disease.

"In conclusion, the treatment should be of a negative character, by which I mean, avoiding everything that may interfere with the natural law of the part affected; or, in other words, let the organism of the intestines effect its own restoration. It is better to keep that principle in view during the treatment; yet, on the other hand, all irritants must be removed, and acute pain must be subdued. In the early stages of the complaint, when the evacuations are of a mucous or muco-fibrinous character, the hydriodate of potash with morphine, may be given with advantage, the former in ten-grain doses night and morning, with half a grain of the latter at bedtime." 208.

